# SIRS criteria as predictors of mortality in patients admitted with sepsis

**DOI:** 10.1186/cc12954

**Published:** 2013-11-05

**Authors:** Adriell Ramalho Santana, Jaqueline Lima de Souza, Fábio Ferreira Amorim, Felipe Bozi Soares, Bárbara Magalhães Menezes, Mariana Pinheiro Barbosa de Araújo, Fernanda Vilas Bôas Araújo, Louise Cristhine de Carvalho Santos, Pedro Henrique Gomes Rocha, Lucila de Jesus Almeida, Thais Almeida Rodrigues, Pedro Nery Ferreira Júnior, Alethea Patrícia Pontes Amorim, José Aires de Araújo Neto, Edmilson Bastos de Moura, Marcelo de Oliveira Maia

**Affiliations:** 1 Escola Superior de Ciências da Saúde, Brasília, Brazil; 2 Liga Acadêmica de Medicina Intensiva de Brasília, Brazil; 3 Hospital Santa Luzia, Brasília, Brazil

## Background

The ACCP/SCCM consensus conference definitions for sepsis are used worldwide [1]. However, consensus definitions do not adequately reflect the intricacies of sepsis and can overestimate the diagnosis due to their high sensitivity. Moreover, the consensus criteria do not allow the staging or the prediction of sepsis outcome [2]. This study aims to evaluate the individual components of SIRS criteria as predictors of mortality in patients admitted to an ICU with sepsis.

## Materials and methods

A case-control study conducted in the ICU of Hospital Santa Luzia, Brasilia, DF, Brazil, during 4 months. Patients were divided into two groups: survivors group (SG) and nonsurvivors group (NSG). The accuracy of individual components of SIRS criteria as predictors of mortality was measured with the area under the receiver operating characteristic (ROC) curve.

## Results

A total of 76 patients were enrolled, 10.5% (*n *= 8) with septic shock. Age was 70 ± 18 years, SAPS3: 52.9 ± 13.9, APACHE II: 15.5 ± 8.8. The ICU length of stay was 9 ± 10 days. ICU mortality was 21% (*n *= 16). The most prevalent sites of infections were respiratory (57.9%, *n *= 44), followed by urinary (25%, *n *= 19) and cutaneous (6.6%, *n *= 5). The incidence of tachycardia was the only parameter higher in the NSG (37.5% vs. 9.1%, *P *= 0.00). There was no difference regarding the incidence of fever or hypothermia (15.4% vs. 24.0%, *P *= 0.38), tachypnea (25.0% vs. 17.5%, *P *= 0.42) or leukocytosis or leucopenia (20.9% vs. 21.2%, *P *= 0.97) between the groups. The relative risk of death in patients with tachycardia was 4.13 (95% CI: 1.46 to 11.63). Tachycardia also had the larger area under the ROC curve: 0.708 (95% CI: 0.566 to 0.850), versus 0.442 (95% CI: 0.287 to 0.597) for fever/hypothermia, 0.556 (95% CI: 0.397 to 0.715) for tachypnea, and 0.498 (95% CI: 0.338 to 0.658) for leukocytosis/leucopenia.

## Conclusions

For this sample, tachycardia was the only SIRS component associated with ICU mortality in patients admitted with sepsis.

## References

[B1] Levy MM Fink MP Marshall JC Abraham E Angus D Cook D Cohen J Opal SM Vincent JL Ramsay G 2001 SCCM/ESICM/ACCP/ ATS/SIS International Sepsis Definitions Conference Crit Care Med 2003 17 1250 1256 10.1097/01.CCM.0000050454.01978.3B 12682500

[B2] Vincent JL Dear SIRS, I'm sorry to say that I don't like you Crit Care Med 1997 17 372 374 10.1097/00003246-199702000-00029 9034279

